# Mild hypothermia effects on serum neuroprotection, nerve growth factor (NGF), brain-derived neurotrophic factor (BDNF), and superoxide dismutase (SOD) levels in neonates with hypoxic-ischemic encephalopathy

**DOI:** 10.5937/jomb0-50866

**Published:** 2025-06-13

**Authors:** Wenfeng Duan, Xuan Wang

**Affiliations:** 1 Northwest Women's and Children's Hospital, Department of Neonatology, Xi'an, Shaanxi Province, China; 2 521 Hospital of Norinco Group, Department of Neonatal, Xi'an, Shaanxi Province, China

**Keywords:** neonatal HIE, levels of cytokines, neurobehavioral score, neonatalna HIE, nivoi citokina, neurobihe - vioralni skor

## Abstract

**Background:**

Neonatal hypoxic-ischemic encephalopathy (HIE) is a serious condition that can lead to long-term neurological damage. Mild hypothermia is a promising treatment for HIE, but its efficacy and safety in newborns are not well established. To evaluate the therapeutic effects of mild hypothermia on neonatal HIE in a randomised controlled trial.

**Methods:**

This was a prospective study of 132 newborns with HIE treated with either mild hypothermia or routine conventional treatment. The primary outcome measures were changes in neural cytokines, brain injury markers, oxidative stress factors, neurological function recovery time, and therapeutic outcomes.

**Results:**

The mild hypothermia group showed significant improvements in neural cytokines (NGF and BDNF), brain injury markers (S100B, NSE, and MBP), and oxidative stress factors (SOD, MDA, IL-18, and caspase-3) compared to the control group. The mild hypothermia group also had a faster neurological function recovery time and a higher total response rate (95.45% vs. 80.30%, P<0.05) compared to the control group.

**Conclusions:**

Mild hypothermia therapy is a safe and effective treatment for neonatal HIE, with significant improvements in neural cytokines, brain injury markers, and oxidative stress factors, as well as faster neurological function recovery time and higher therapeutic outcomes. Results: The mild hypothermia group showed significant improvements in neural cytokines (NGF and BDNF), brain injury markers (S100B, NSE, and MBP), and oxidative stress factors (SOD, MDA, IL-18, and caspase-3) compared to the control group. The mild hypothermia group also had a faster neurological function recovery time and a higher total response rate (95.45% vs. 80.30%, P<0.05) compared to the control group. Conclusions: Mild hypothermia therapy is a safe and effective treatment for neonatal HIE, with significant improvements in neural cytokines, brain injury markers, and oxidative stress factors, as well as faster neurological function recovery time and higher therapeutic outcomes. The novelty of this work was that it showed potential biomarkers for evaluating response to treatment and the pathophysiological effect of treatment by assessing these biomarkers.

## Abstract (sr)

**Uvod:**

Neonatalna hipoksično-ishemijska encefalopatija (HIE) kod novorođenčadi predstavlja ozbiljno stanje koje može dovesti do dugotrajnog neurološkog oštećenja. Blaga hipotermija je obećavajući tretman za HIE, ali njena efikasnost i bezbednost kod novorođenčadi još nisu dovoljno potvrđene. Procena terapeutskih efekata blage hipotermije na hipoksično-ishemijsku encefalopatiju kod novorođenčadi u randomizovanoj kontrolisanoj studiji.

**Metode:**

Ovo je bila prospektivna studija na 132 novorođenčeta sa HIE koja su bila lečena bilo blagom hipotermijom ili rutinskim konvencionalnim tretmanom. Primarne mere ishoda bile su promene neuralnih citokina, markera povrede mozga, faktora oksidativnog stresa, vreme oporavka neurološke funkcije i terapeutski ishodi.

**Rezultati:**

Grupa sa blagom hipotermijom pokazala je značajna poboljšanja u nivou neuralnih citokina (NGF i BDNF), markera povrede mozga (S100B, NSE i MBP) i faktora oksidativnog stresa (SOD, MDA, IL-18 i kaspaza-3) u poređenju sa kontrolnom grupom. Grupa sa blagom hipo termijom tako|e je imala brže vreme oporavka neurološke funkcije i veću ukupnu stopu odgovora na lečenje (95,45% naspram 80,30%, P<0,05) u poređenju sa kontrolnom grupom.

**Zaključak:**

Terapija blagom hipotermijom je bezbedan i efikasan tretman za hipoksično-ishemijsku encefalopatiju kod novoro|enčadi, sa značajnim poboljšanjima u neuralnim citokinima, markerima povrede mozga i faktorima oksidativnog stresa, kao i bržim oporavkom neurološke funkcije i boljim terapijskim ishodima. Novina ovog istraživanja je u tome što pokazuje potencijalne biomarkere za procenu odgovora na lečenje i patofizioločki efekat tretmana kroz analizu ovih biomarkera.

## Introduction

Neonatal hypoxic-ischemic encephalopathy (HIE) is common in premature infants or perinatal asphyxia. Due to insufficient oxygen supply to the brain, the brain is seriously damaged, which may lead to infant death or permanent neurological dysfunction, and the prevalence of the disease is increasing year by year [1]. Neonatal HIE can produce sequelae such as motor disorders, cognitive dysfunction, epilepsy, mental retardation, hearing or vision impairment [2]. The legacy of the trigger for the baby causes a long-term impact on quality of life (QoL) and development. Through in vivo and in vitro and randomised controlled clinical research, it was found that mild hypothermia therapy on neonatal HIE has an apparent curative effect, which is simple and safe [3]. However, due to a variety of factors, some children missed the best treatment opportunity, resulting in the effect of mild hypothermia therapy in the clinical treatment of such children with significant differences. Therefore, neonatal HIE and mild hypothermia therapy research is still a necessity, and further exploring the pathogenesis of the disease and treatment is of vital importance to improve the survival rate and QoL of the baby.

The pathophysiology of neonatal HIE is very complex. Through in-depth research, researchers have made great progress in understanding neonatal HIE’s pathogenesis. Studies have shown that in the primary stage of brain injury, there will be oxidative metabolism suspension, anoxic depolarisation, cell necrosis, and cytotoxic oedema. When the blood flow and oxygen supply system are normally operating, there will be transient normal regulation of oxidative metabolism, EEG inactivity, hypoperfusion, and other phenomena. During this period, children will experience loss of oxidative metabolism, secondary cell swelling, vascular damage, and other phenomena, and may also occur epilepsy. Cell injury and death in neonatal HIE mostly happen in the secondary period; continuous cell death, apoptosis, and cell autophagy occur, leading to persistent inflammation [4] [5] [6] [7]. Neuroinflammation occurs in blood vessels, followed by damage to brain parenchymal cells, the activation of astrocytes and microglia and the infiltration of peripheral immune cells in the brain, further aggravating the inflammatory response in blood vessels [8]. Perinatal brain injury caused by ischemia/hypoxia and peripheral immune system stimulation leads to the activation of different immune cell subsets and brain infiltration. Currently, the effectors of peripheral immune cells include the formation of neutrophil extracellular network structures (NETs), reactive oxygen species (ROS) production, inflammation, increased activity of basement membrane degradation enzymes (COX-2 and MMP), and the release of pro-inflammatory cytokines [9]. Studies have found that neuroinflammation after hypoxia-ischemia triggered by innate and adaptive immune responses leads to brain vulnerability [10]. Studies show that inflammation in the pathogenic process of HIE plays a key role in early or late brain injury and brain tissue repair [11].

Mild hypothermia therapy is a widely used clinical treatment at present. Studies have found that mild hypothermia therapy is very effective in the neuroprotective effect of neonatal HIE [12]. This treatment reduces the degree of brain damage by reducing the child’s body temperature to a lower temperature below normal body temperature [13]. Cooling the body temperature to 33–34°C has been shown to improve neurodevelopmental outcomes in neonates with hypoxic-ischemic encephalopathy by reducing brain injury and inflammation [14]. A multicenter randomised controlled trial demonstrated that mild hypothermia therapy initiated within 6 hours of birth in neonates with HIE resulted in significant reductions in mortality and disability at 18–22 months of age [15].

It has been proposed that hypoxia-ischemia can activate nuclear factor erythroid 2-related factor 2 (Nrf2) expression and related pathways. It results in the reduction of oxidative products ROS and malondialdehyde (MDA), as well as the levels of cytokines tumour necrosis factor-α (TNF-α), interleukin-6 (IL-6), and high mobility group box 1 (HMGB1), and the upregulation of levels of antioxidant enzymes superoxide dismutase (SOD), glutathione peroxidase (GPx), and catalase (CAT) [16]. In addition, some studies have suggested that feeding children during mild hypothermia therapy does not increase the risk of necrotising enterocolitis, hypoglycemia or feeding intolerance and can reduce the incidence of sepsis and all-cause mortality [17].

Studies of treatment and neuroprotective mechanisms are important to prevent infant death and reduce disability rates. As an effective treatment, mild hypothermia therapy plays a major role in neuroprotection by regulating cell metabolism, reducing inflammatory response, and affecting cytokine levels. This article aimed to explore the effect of mild hypothermia therapy on neonatal HIE, which helps improve its prognosis and clinical treatment level.

## Materials and methods

This was a prospective study of a total of 132 newborns with HIE who were treated with either mild hypothermia or routine, conventional treatment. This study was conducted from January 2022 to April 2023 at the Northwest Women’s and Children’s Hospital. Simple avaia=labe sampling was performed on all eligible patients for the study.

### Inclusion criteria

To be eligible for the study, participants had to meet the following criteria:

1. Diagnosed with HIE by a paediatrician at the Northwest Women’s and Children’s Hospital using established diagnostic criteria [18].
2. Admitted to the hospital within 12 hours of birth.
3. Gestational age between 37 weeks (259 days) and 42 weeks (293 days).
4. Birth weight greater than 2.5 kilograms.

### Exclusion criteria

Participants were excluded from the study if they had any of the following conditions:

1. Severe congenital malformations or congenital heart disease.
2. Intracerebral hemorrhage.
3. Serious infectious diseases.
4. Severe bleeding or other haematological disorders.
5. Other severe neonatal illnesses.

### Ethics and informed consent

This study was approved by the Ethics Committee of the Northwest Women’s and Children’s Hospital (approval number: K91802N1293). Written informed consent was obtained from all participants’ parents or legal guardians before enrollment. The study was conducted following the principles of the Declaration of Helsinki and the Guidelines for Good Clinical Practice.

### Sample size calculation

The sample size was calculated based on a previous study [18] that reported a significant difference in outcome between mild hypothermia and control groups. Assuming a two-sided test with a significance level of 0.05 and a power of 80%, we estimated that 132 participants (66 per group) would be required to detect a clinically significant difference in the primary outcome measure.

### Treatment methods

The controls adopted conventional treatment. Treatment methods: Maintaining normal ventilation and respiration, normal operation of brain and systemic blood flow perfusion, and normal blood sugar status of patients, reducing intracranial pressure and other comprehensive treatments for intervention. An intravenous infusion of 1L 10% glucose+5 mL cerebrolysin was applied for treatment, with each infusion not exceeding 3 hours once a day [19].

The mild hypothermia group was treated with mild hypothermia therapy based on conventional treatment. Treatment methods: On the rescue table the newborn was placed on a far-infrared radiant rescue table with its skin exposed and away from any heating facilities. Before treatment, relevant blood biochemical tests were required, and a rectal temperature probe was inserted approximately 5 cm into the rectum and fixed to the side of the thigh. A temperature probe was placed on the abdomen to monitor the skin temperature. When cooling was initiated, induction needed to be performed rapidly to ensure the target temperature reached within 1 to 2 hours. In the process of treatment, the rectal temperature was 32°C–34°C, a maximum range of 33°C to 34.5°C. Seventy-two hours after treatment, natural thawing can be performed, and when necessary, far infra red rewarming is carried out. Typically, the rewarming rate was increased by 1°C every 2 hours, and the time to return to normal temperature must last more than 6 hours. During mild hypothermia therapy, it was necessary to closely observe the neurological manifestations of neonates and dynamically monitor blood pressure, respiration, electrolytes, and coagulation. If severe adverse reactions occur during the treatment, mild hypothermia therapy should be stopped immediately. Through remedy, the newborn’s condition should be closely observed for at least 24 hours, and the treatment should be continued for 4 weeks.

### Indicators of observation

Venous peripheral blood was collected from the children before remedy and on day 14 of treatment. A total of 3 mL of peripheral blood was collected, and the supernatant was collected by centrifugation and stored at -80°C for detection.

The levels of neural cytokines of the subjects were compared. The levels of nerve growth factor (NGF), brain-derived neurotrophic factor (BDNF), S100 calcium-binding protein B (S100B), myelin basic protein (MBP), and neuron-specific enolase (NSE) were detected by double-antibody sandwich enzyme-linked immunosorbent assay (ELISA). The operations were carried out strictly based on the instructions.

The levels of oxidative stress factors in the subjects’ serum were compared. The level of SOD in the serum and the levels of MDA, IL-18, and Caspase-3 were detected by ELISA.

### Neonatal behavioural neurological measurement

The recovery time of neurological function and neurobehavioral scores were compared. The neonatal behavioural neurological assessment (NBNA) scale was adopted to evaluate the integrity of neonatal nervous system development [20]. The NBNA scale assessed neonatal behaviour ability, active and passive muscle tension muscle tension, primitive reflex, and general valuation in five aspects, 20 items. Professional doctors evaluated all children at 3 d, 7 d, 10 d, 14 d, 17 d, 21 d, 24 d, and 28 d, a total of 40 points. Thirty-five points or above was considered as normal neurological function and less than 35 points as abnormal neurobehavioral function.

### Therapeutic outcome evaluation standard

The effect of mild hypothermia therapy was evaluated from the symptoms, signs, and neurobehavioral function scores of neonatal HIE. The treatment was markedly effective when the clinical symptoms disappeared, the signs returned to normal, and the NBNA score was more than 35. The treatment was effective if the clinical symptoms and signs were improved and the NBNA score was more than 30. The treatment was ineffective if the clinical symptoms and signs were unchanged or more severe and the NBNA score was less than 30.

### Statistical analysis

SPSS24.0 data analysis software was adopted to process all data; measurement data were expressed by mean ± standard deviation (±s), and a t-test was adopted. Count data were expressed by percentage (%) and compared by χ^2^ tests. Independent t-tests among groups compared continuous data, and repeated measure ANOVA was performed to compare changes in metric variables during the time. The difference was statistically considerable, with *P*<0.05.

## Results

Controls had 37 male children and 29 female children; the mean age was (37.83±2.14) weeks, and the average weight was (3.41±0.52) kg, 21 mild HIE children, 28 moderate HIE children, 17 severe HIE children. There were 38 male and 28 female children in the mild hypothermia group, with an average age of (37.74±2.09) weeks and an average weight of (3.48±0.49) kg. There were 20 children with mild HIE, 26 with moderate HIE, and 20 with severe HIE (Table 1). There was no obvious difference in age, gender, and disease severity between the two groups (*P*>0.05).

**Table 1:** Characteristics of Study Participants and Outcome Measures. * Indicates a significant difference between pre-remedy and post-remedy valueswithin the same group.

Variable	Control Group (n=66)	Mild Hypothermia Group (n=66)	P-value
General Information			
Male/Female	37/29	38/28	0.83
Age (weeks)	37.83±2.14	37.74±2.09	0.71
Weight (kg)	3.41±0.52	3.48±0.49	0.53
HIE Severity			
Mild	21	20	0.83
Moderate	28	26	0.76
Severe	17	20	0.56
Neural Cytokines			
NGF (pre-remedy)	12.5±3.5	12.2±3.8	0.74
NGF (post-remedy)	12.5±3.5	15.6±4.2*	<0.05
BDNF (pre-remedy)	8.5±2.5	8.2±2.8	0.73
BDNF (post-remedy)	8.5±2.5	11.4±3.1*	<0.05
Brain Injury Markers			
S100B (pre-remedy)	120.4±25.6	118.2±24.1	0.71
S100B (post-remedy)	120.4±25.6	90.5±20.1*	<0.05
NSE (pre-remedy)	35.6±10.3	34.5±9.8	0.73
NSE (post-remedy)	35.6±10.3	25.6±8.5*	<0.05
MBP (pre-remedy)	250.1±50.6	245.9±49.2	0.76
MBP (post-remedy)	250.1±50.6	190.3±40.5*	<0.05
Oxidative Stress Factors			
SOD (pre-remedy)	80.2±15.6	78.5±14.9	0.74
SOD (post-remedy)	80.2±15.6	95.1±18.2*	<0.05
MDA (pre-remedy)	2.5±0.8	2.4±0.7	0.83
MDA (post-remedy)	2.5±0.8	1.8±0.5*	<0.05
IL-18 (pre-remedy)	120.5±25.1	118.9±23.6	0.76
IL-18 (post-remedy)	120.5±25.1	90.2±20.3*	<0.05
Caspase-3 (pre-remedy)	35.9±10.5	34.8±9.9	0.74
Caspase-3 (post-remedy)	35.9±10.5	25.9±8.1*	<0.05

The study compared the characteristics and outcome measures of 66 participants in a control group with 66 participants in a mild hypothermia group. The two groups were similar in demographics, with no significant differences in sex ratio, age, or weight. The severity of HIE was also comparable between the two groups, with mild, moderate, and severe cases distributed evenly.

After treatment, the study found significant changes in various biomarkers and oxidative stress factors in the mild hypothermia group. Specifically, the levels of neural cytokines NGF and BDNF increased significantly, while brain injury markers S100B, NSE, and MBP decreased significantly. Additionally, the levels of oxidative stress factors SOD and MDA changed significantly, with SOD increasing and MDA decreasing. The levels of IL-18 and caspase-3 were also substantially reduced in the mild hypothermia group after treatment. These changes were not observed in the control group, suggesting that mild hypothermia may have a beneficial effect on the outcome of patients with HIE.

### NBNA score

**Figure 1:**
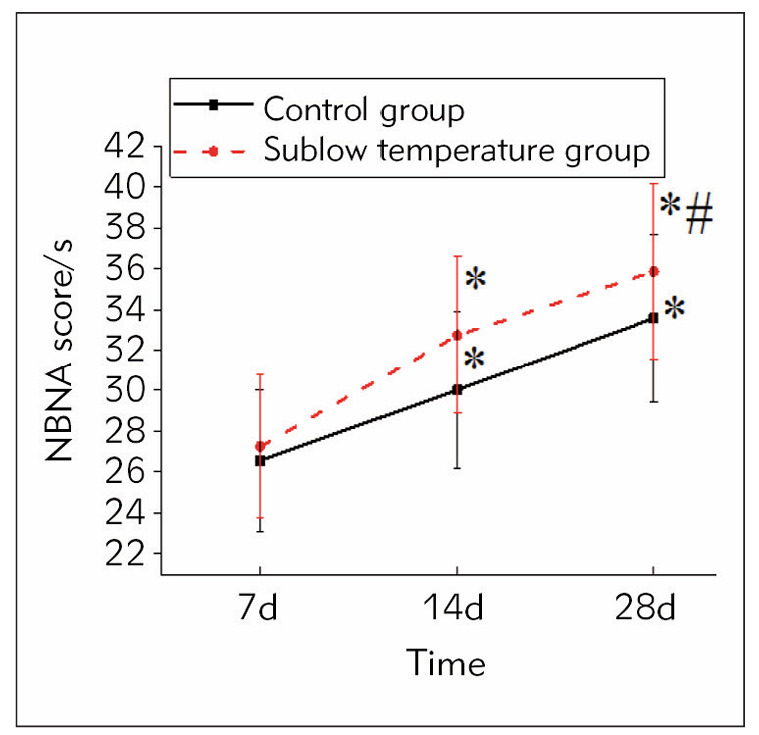
Contrast of NBNA scores in subjects.<br>Note: As against the 7^th^ day of intervention, *P<0.05; As against controls, #P<0.05

Figure 1 illustrates that on the 7th day of the intervention, the NBNA score of the mild hypothermia group was slightly higher than in controls (*P*>0.05). Through 14 d of intervention, the NBNA score of the controls was clearly higher than that at 7 d of intervention (*P*<0.05). The NBNA score of mild hypothermia therapy-treated subjects was markedly higher than the controls (*P*<0.05). Through 28 d of intervention, the neurological function of the mild hypothermia therapy-treated subjects returned to normal. At the same time, the NBNA score of the controls increased visibly but did not return to normal.

### Neurological function recovery time of subjects

**Figure 2:**
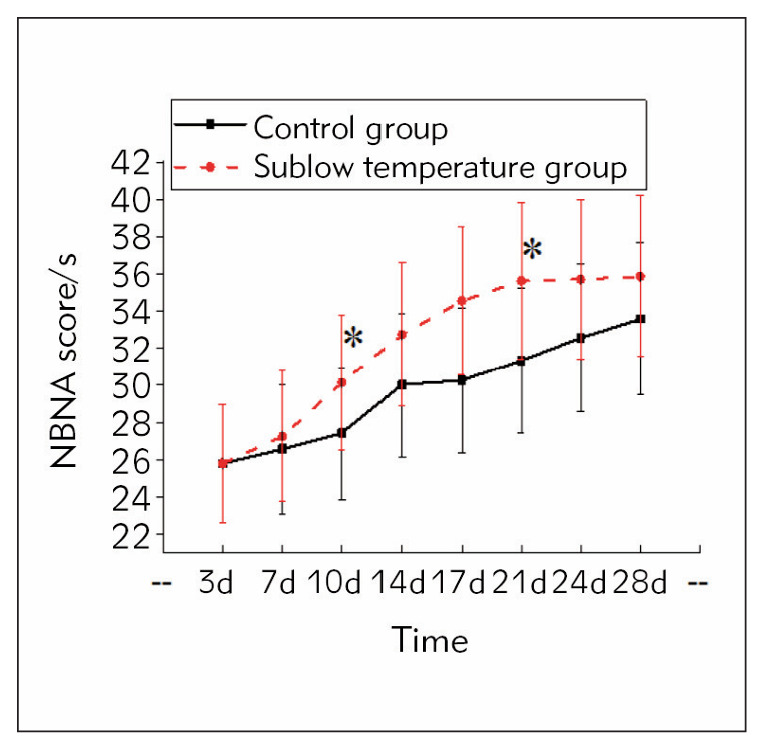
Contrast of neurological function recovery time of subjects.<br>Note: As against controls, *P<0.05

Figure 2 illustrates that before 7 d of treatment, the NBNA score of the mild hypothermia group was slightly higher than in controls (*P* >0.05). The neurological function of the controls recovered effectively following 14 d of intervention, and the mild hypothermia therapy-treated subjects recovered effectively following 10 d of intervention. The neurological function of the mild hypothermia therapy-treated subjects recovered to normal at 21 d of intervention. In comparison, the neurological function of the controls did not recover to normal at the end of 28 d of treatment, indicating that the effective recovery time of the mild hypothermia therapy-treated subjects was visibly shorter than in controls (*P*<0.05).

### Efficacy of subjects

**Figure 3:**
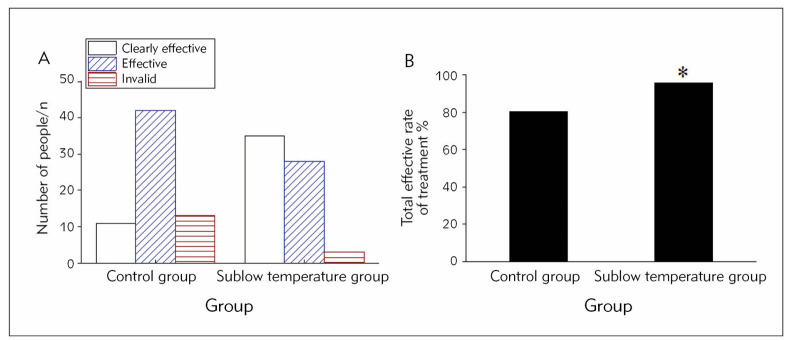
Contrast of therapeutic outcomes in subjects (A is the number of patients with different therapeutic outcomes, B is the total response rate).<br>Note: As against controls, *P<0.05

Figure 3 illustrates that in the mild hypothermia group, 35 cases (53.03%) were visibly effective, 28 cases (42.42%) were effective, and 3 cases (4.54%) were ineffective. In the controls, 11 cases (16.67%) were visibly effective, 42 cases (63.63%) were effective, and 13 cases (19.70%) were ineffective. The results suggested that the total response rate of mild hypothermia therapy-treated subjects (95.45%) was visibly superior as against controls (80.30%) (*P*<0.05).

## Discussion

Neonatal HIE is mainly caused by insufficient blood transfusion and insufficient oxygen supply to the brain. Hypoxia-ischemia may lead to the death of neurons. Several studies have investigated the effects of therapeutic hypothermia on neonatal HIE. Azzopardi et al. [21], Shankaran et al. [14], Gluckman et al. [15], and Zhou et al. [22] conducted randomised controlled trials that demonstrated the efficacy of hypothermia in reducing the risk of death or severe disability in infants with HIE. While the studies showed similar outcomes, they differed in their measurements, with some assessing brain injury markers, neural cytokines, and oxidative stress factors. The studies by Azzopardi et al. [21] and Gluckman et al. [15] focused on the primary outcome of death or severe disability, while Shankaran et al. [14] and Zhou et al. [22] also measured brain injury improvement as well as our study. However, our study is notable for its comprehensive measurement of neural cytokines, brain injury markers, oxidative stress factors, and neurological function recovery time. It provides insight into the underlying mechanisms of hypothermia’s neuroprotective effects. We found that the mild hypothermia group showed significant improvements in neural cytokines (NGF and BDNF), brain injury markers (S100B, NSE, and MBP), and oxidative stress factors (SOD, MDA, IL-18, and caspase-3) compared to the control group.

Studies have found that reducing mitochondrial membrane potential, adenosine triphosphate (ATP), and the excessive production of intracellular lactic acid can induce primary neuronal death. The secondary energy failure of neurons will lead to secondary neuronal death [23]. Neonatal HIE can lead to increased inflammatory response and apoptosis of nerve cells, oxidative stress is an essential cause of brain damage in children, and oxygen-free radical reaction can lead to neuronal damage [24]. The best time for mild hypothermia therapy is within 6 hours after birth, but some studies have shown that the scheme is still effective when the time is delayed from 6 to 12 hours [25]. Therefore, the inclusion criteria in this article are patients admitted within 12 hours after birth. After neonatal ischemia and hypoxia, innate immunity is activated immediately. Oxidative stress and neuronal death can activate glial cells to release inflammatory factors and ROS, which participate in cell apoptosis. The activation of glial cells also promotes the release of metalloproteinases, pro-inflammatory cytokines, and chemokines, which recruit and activate immune cells. These cells release nitric oxide (NO), SOD, and inflammatory cytokines, which lead to the death of neurons.

Other interventions are also thought to help neuroprotection in HIE. For example, Zhu et al. [26] found that erythropoietin (Epo) improved neurologic outcomes in newborns with HIE. Aly et al. [27] reported that melatonin use for neuroprotection in perinatal asphyxia was associated with improved outcomes. Additionally, Ahmed et al. [28] highlighted the potential of melatonin for neuroprotection in preterm brain injury in a review of preclinical evidence. Further more, Barata et al. [29] demonstrated that cannabidiol and hypothermia provided neuroprotection in a piglet model of newborn hypoxic-ischemic brain damage. In the same manner, mild hypothermia therapy can reduce the metabolic rate of the brain and the production of cytotoxic substances in neonates with HIE, thus playing a neuroprotective role. Studies have found that neuron apoptosis, ROS level, and MDA content in the ischemia-reperfusion injury rat model increased visibly.

After fentanyl was given, neuron apoptosis, neuron ROS level, and MDA content of the rat decreased clearly [30]. In addition, it has been found that mild hypothermia therapy has a clear increase in NBNA scores in clinical practice [31]. This is consistent with the results of this article, in which the neurological function of the children was visibly improved after the intervention of mild hypothermia. NSE is an acidic glycolytic enzyme of neuroendocrine cells and a marker of neuronal damage such as epilepsy. When the blood-brain barrier is destroyed, NSE will be released from brain cells, resulting in increased levels of NSE in the intercellular space and cerebrospinal fluid, which enter the blood circulation so that high levels of NSE can be detected in serum. Some studies have also found that the concentration of serum NSE is related to the increase of intracranial pressure and the decrease of perfusion pressure. The level of NSE increases with the increase of cerebral hematoma, so the level of NSE significantly impacts the volume of cerebral hematoma, brain oedema, and prognosis recovery [32]. In this article, the level of NSE in the serum of HIE children was visibly increased, decreased in the subjects following remedy, and decreased more markedly in the mild hypothermia therapy group. BDNF mainly exists in the amygdala, hippocampus, and cerebral cortex, which can promote the growth and development of the central nervous system and regulate the activity of neurons. BDNF is more active in infant development, and its change trend decreases as the nervous system develops and matures in adulthood. In the hypoxic and ischemic rat models, the expression level of BDNF in the cortex and hippocampus is decreased. In this article, the expression level of BDNF is up-regulated, the expression of voltage-dependent anion-selective channel 1 (VDAC-1) is reduced, and the expression of syntaxin 1B (STX1B) is increased, thus promoting the regeneration of neurons’ axons [33]. In this article, the serum BDNF level of HIE children was markedly decreased. It markedly increased the following remedy, and the BDNF level of patients with mild hypothermia therapy increased more. These results suggest that BDNF, a neuroprotective agent, can reduce neuronal damage and stimulate axon cell proliferation and synaptic sensitivity.

Studies have found that in the HIE rat model, the level of NGF was markedly lower, and sevoflurane can raise it. In this article, the level of NGF in the serum of HIE children was decreased. Following remedy, it was found that the level of NGF in subjects was markedly increased, which was increased more markedly in the hypothermia treatment group. An in vitro study has found that the miR-579-3p/TRAF6 axis pathway is involved in HIE. SOD levels were decreased, and MDA content was increased in an in vitro HIE cell model. The inhibition of miR-579-3p suppressed the enhancement of SOD activity and the down-regulation of MDA level induced by circ_0007706 deficiency [34]. The cryogenic treatment can promote the rise of the level of SOD and MDA level.

### Limitations

This study is not without its limitations, which primarily stem from its retrospective design, potentially introducing biases in patient selection and data collection, thereby compromising the internal validity of the findings. Furthermore, the relatively small sample size of 132 newborns may limit the generalizability of the results, rendering it uncertain whether the observed effects can be extrapolated to a broader population of neonates with hypoxic-ischemic encephalopathy.

## Conclusion

Mild hypothermia therapy has an obvious neuroprotective outcome on neonatal HIE. By regulating the levels of cytokines, mild hypothermia therapy can reduce the degree of nerve injury and promote the recovery of nerve function. The up-regulation of neuroprotective factors such as NGF and BDNF may be one of the critical mechanisms of mild hypothermia therapy. These results contribute to a deeper understanding of the mechanism of mild hypothermia therapy in neonatal HIE and provide new ideas for clinical treatment. The novelty of this work lies in its pioneering demonstration of the potential utility of specific biomarkers as valuable tools for evaluating the response to treatment, thereby providing a more nuanced understanding of the therapeutic efficacy of mild hypothermia in neonatal hypoxic-ischemic encephalopathy. Moreover, this study has also shed light on the underlying pathophysiological effects of treatment by comprehensively assessing the dynamics of these biomarkers, thereby offering a more complete picture of the complex interplay between the therapeutic intervention and the biological processes involved in this debilitating condition, ultimately contributing to the development of more effective and targeted treatment strategies.

## Dodatak

### Conflict of interest statement

All the authors declare that they have no conflict of interest in this work.
